# Enhancing CAR T cells function: role of immunomodulators in cancer immunotherapy

**DOI:** 10.1007/s10238-024-01442-9

**Published:** 2024-08-06

**Authors:** Maheen Rehman, Ariba Qaiser, Hassan Sardar Khan, Sobia Manzoor, Javed Ashraf

**Affiliations:** 1 Molecular Virology Lab, Atta-Ur-Rahman School of Applied Biosciences, National University of Sciences and Technology, Islamabad, Pakistan; 2 Institute of Dentistry, University of Eastern Finland, Kuopio, Finland; 3 Riphah International University, Islamabad, Pakistan

**Keywords:** CAR T cells, Combinatory therapy, Immunotherapy, Immunomodulators, Cancer

## Abstract

CAR T-cell therapy is a promising immunotherapy, providing successful results for cancer patients who are unresponsive to standard and traditional therapeutic approaches. However, there are limiting factors which create a hurdle in the therapy performing its role optimally. CAR T cells get exhausted, produce active antitumor responses, and might even produce toxic reactions. Specifically, in the case of solid tumors, chimeric antigen receptor T (CAR-T) cells fail to produce the desired outcomes. Then, the need to use supplementary agents such as immune system modifying immunomodulatory agents comes into play. A series of the literature was studied to evaluate the role of immunomodulators including a phytochemical, Food and Drug Administration (FDA)-approved targeted drugs, and ILs in support of their achievements in boosting the efficiency of CAR-T cell therapy. Some of the most promising out of them are reported in this article. It is expected that by using the right combinations of immunotherapy, immunomodulators, and traditional cancer treatments, the best possible cancer defying results may be produced in the future.

## Introduction

Chimeric antigen receptors are synthetically designed recombinant receptors. They bind to specific antigens and perform T-cell activating functions. CARs are living drugs that have a great aptitude for cancer immunotherapy [1]. Chimeric antigen receptor T cells (CAR-T cells) are T cells that are expanded after stimulation and have genetic modifications. T cells are extracted from a donor’s blood and after their conversion into CAR T cells, they are infused into the subjects of research, clinical trial, or a patient undergoing CAR T-cell immunotherapy [2]. Immunotherapy of the CAR T type may be autologous or allogenic [2, 3]. The genetically modified T cells begin to express a chimeric antigen receptor (CAR) which targets an antigen of neoplastic origin [4]. CAR-T cell therapy has achieved the status of a hallmark therapeutic approach for hematological malignancies. Currently, six CAR-T products have secured the approval of the Food and Drug Administration (FDA) for treating lymphoma, leukemias, & multiple myeloma [5] The scope of CAR T-cell therapy beyond oncology is for the treatment of infectious as well as autoimmune diseases [6].

Although clinical trials of CAR T cells have been successful for liquid malignancies for both pediatric and non-pediatric patients, major side effects have been observed. Some of the side effects reported in the literature, with an incidence greater than 20% are hypogammaglobulinemia, sepsis, encephalopathy, bleeding episodes, coagulopathy, hypotension, tachycardia, viral infectious disorders, and hypoxia [7–14]. In addition to side effects, CAR T-cell therapy faces several limiting factors. Tumors can develop resistance to CAR T cells targeting an antigen exclusively. For example, targeting solid tumor-specific antigens like human epidermal growth factor receptor 2 (HER2) can result in off-tumor toxicity as these antigens are also present on normal tissues. CAR T cells may also fail to infiltrate solid tumors and can become exhausted during the process [15]. Limitations of CAR T cells are further discussed in (Fig. 4) of this article.

To counteract the potential limitations of (Fig. 4) CAR T-cell therapy, it is essential to enhance their action through combinatory medicinal approaches. This approach not only improves the efficiency of immune function but also mitigates immune toxicity-related reactions [16–19]. The use of immunomodulators is one such combinatory approach in CAR T-cell therapy. Historically, immunomodulators are known as medicines that stimulate our immune system to work more effectively. Immunomodulators are phytochemicals, small molecules, cytokines, interleukins, chemicals derived from microbes or adjuvants, and even targeted drugs. Immunomodulators can manipulate and regulate the immune system to enhance CAR T-cell function [16, 18, 20]. The immune modulators alleviate or over-activation caused by cancer. They are also prestigious as most patients tolerate them more than traditional therapies. Conversely, in some rare cases, patients may have innate or acquired resistance to certain immunomodulators [21].

In this review, we will focus on the mechanistic functioning of the immune modulators, and how they enhance CAR T-cell therapy in fighting cancer. The review presents an organized discussion of some immunomodulators, i.e., sulforaphane, sunitinib, sorafenib, dasatinib, metformin, and IL-23 and IL-15 aiding CAR T-cell therapy specifically in the mitigation of cancer. This article backs the debate as to why combinatory therapies are better suited than a single immune therapies. Aggressive tumor microenvironment (TME), molecular heterogeneity, and a lack of effective combinatory therapies are major hurdles to the oncogenic prognosis. Combinatory approaches in oncological therapeutics augment CAR T cells therapy by 1—strengthening infiltration capability, 2—preserving physiology in harsh TMEs, 3—overcoming antigenic escape, and 4—providing safer compliments that promise lowered vulnerability of patients to CAR T cells associated side effects [22].

## Structure and production of CAR T cells

The CAR in its structure consists of four regions or domains. All of these play a distinct role in the function of CAR T cells. These are ectodomain, hinge domain, transmembrane domain, and endo-domain. The ectodomain, also called extracellular domain, is the region containing a membranous protein located outside the CAR T cell’s cytoplasm exposed into the extracellular space. This domain includes an antigen recognition region, a signaling peptide [also called the antigen-binding domain], and a linker [15, 23, 24]. The signaling peptide consists of a single-chain variable fragment (scFv) formed by linking the variable heavy and light chains of an immunoglobulin through a flexible linker made of amino acids such as serine and glycine. When the antigen-binding domain binds to the tumor-associated antigen (TAA), activation of CAR T cells occurs in a major histocompatibility complex (MHC)-independent fashion, although some CAR T cells can activate in an MHC-dependent manner [15].

The hinge domain connects the ectodomain to the transmembrane domain. Historically, CAR T cells used a spacer region, but the hinge domain was found to be crucial for providing flexibility and length to the CAR. This additional length brings antigens, such as mesothelin, closer to the CAR T-cell membrane, enhancing their efficacy. CAR T cells with a hinge region also show better proliferation compared to those without it [25]. The hinge domain typically consists of amino acid fragments from IgG1, CD8α, and IgG4, with IgG4 being the most promising candidate [26].

The transmembrane domain comprises of an alpha helix which is hydrophobic in nature. It traverses the membrane anchoring the CAR into the T-cell membrane [4]. The integrity of the CAR is dependent on the transmembrane domain [27, 28]. Studies also suggest that transmembrane domain effects CAR expression level, stability, and immuno-synapse formation. The transmembrane domain of the CAR T cells is extracted from the transmembrane domain of CD8, CD3, FcεRI, or CD28 [29].

The endo-domain, or intracellular domain, is the physiological end of the receptor. The most common feature of this domain is CD3*ζ*, which includes three immune receptor tyrosine-based activation motifs (ITAMs) [29]. After the antigen is recognized and bound, the signal is activated, and is transmitted to the CAR T-cell. In short, mechanistically endo-domain engages in signal transduction. Since the development of CARs in 1989, endo-domain has been a key factor in the engineering of CAR T cells [26, 29].

**Fig. 1:**
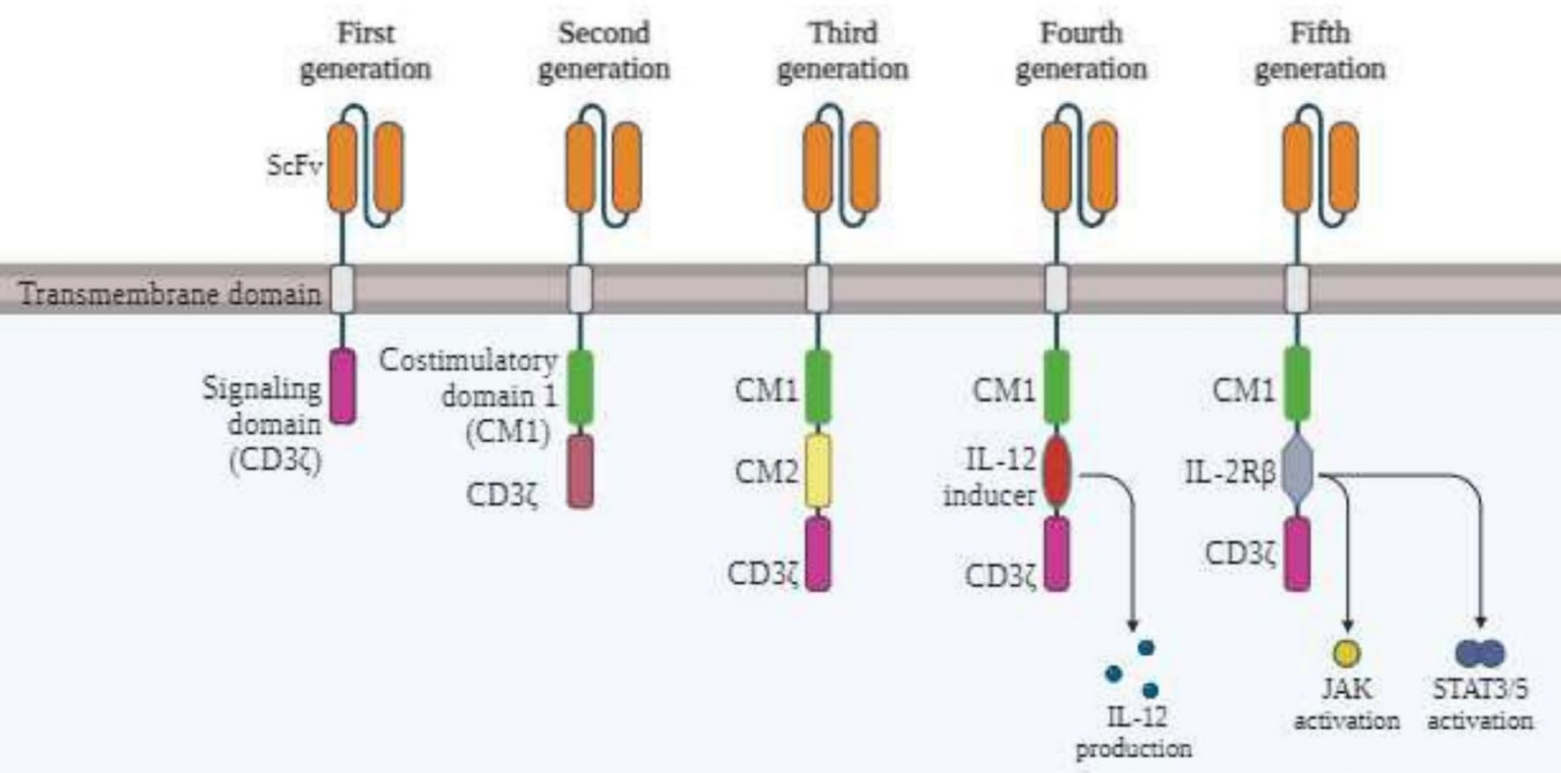
**“Evolution of CARs.”** CAR-T cells in their first generation contained heavy and light chains in the variable regions of their ectodomain, along with a CD3z endo-domain. They kept evolving to have more co-stimulatory domains in their endo-domain. The fourth generation of CAR T cells contained an extra IL-12 producing domain in their intracellular region. In CAR-T cells of the fifth generation, another fragment is added to activate STAT3 signaling, promoting persistence and proliferation

Currently, there are five generations of CAR-T cells [26] [Fig. 1]. The CARs in their first generation had only one signaling domain, usually CD3 zeta or FcR*γ*, leading to reduced effectiveness and limited IL-2 production which is necessary of successful activation of T cells [30, 31]. Addressing this second generation of CARs included a co-stimulatory molecule such as CD28 or B7 which are FDA-approved, promoting IL-2 synthesis and high patient response rate. The co-stimulatory signal ensures that dual-signal requirement for CAR-T cell activation is fulfilled. The CARs of the third generation incorporate multiple signaling domains, such as CD3ζ-CD28-OX40 or CD3*ζ*-CD28-41BB, augmenting the killing capacity of the CAR T cells and cytokine production [32]. They are constructed by adding interleukin-12 (IL-12) to the second-generation constructs. The fourth-generation CARs, also known as T cells, redirected for universal cytokine-mediated killing (TRUCKs), add IL-12 to the second-generation constructs, combining co-stimulatory and pro-inflammatory domains to prevent antigen escape, a significant limitation of earlier CAR T cells [33]. The fifth generation of CARs have been engineered recently, which are further improving the therapeutic efficacy of the CAR-T cells. Structurally, it has an additional fragment of IL2RB chain, located intracellularly, which binds to STAT3, causing signal activation. Better persistence of the fifth generation CAR T cells is ensured by promoting proliferation and preventing terminal differentiation [34].

**Fig. 2:**
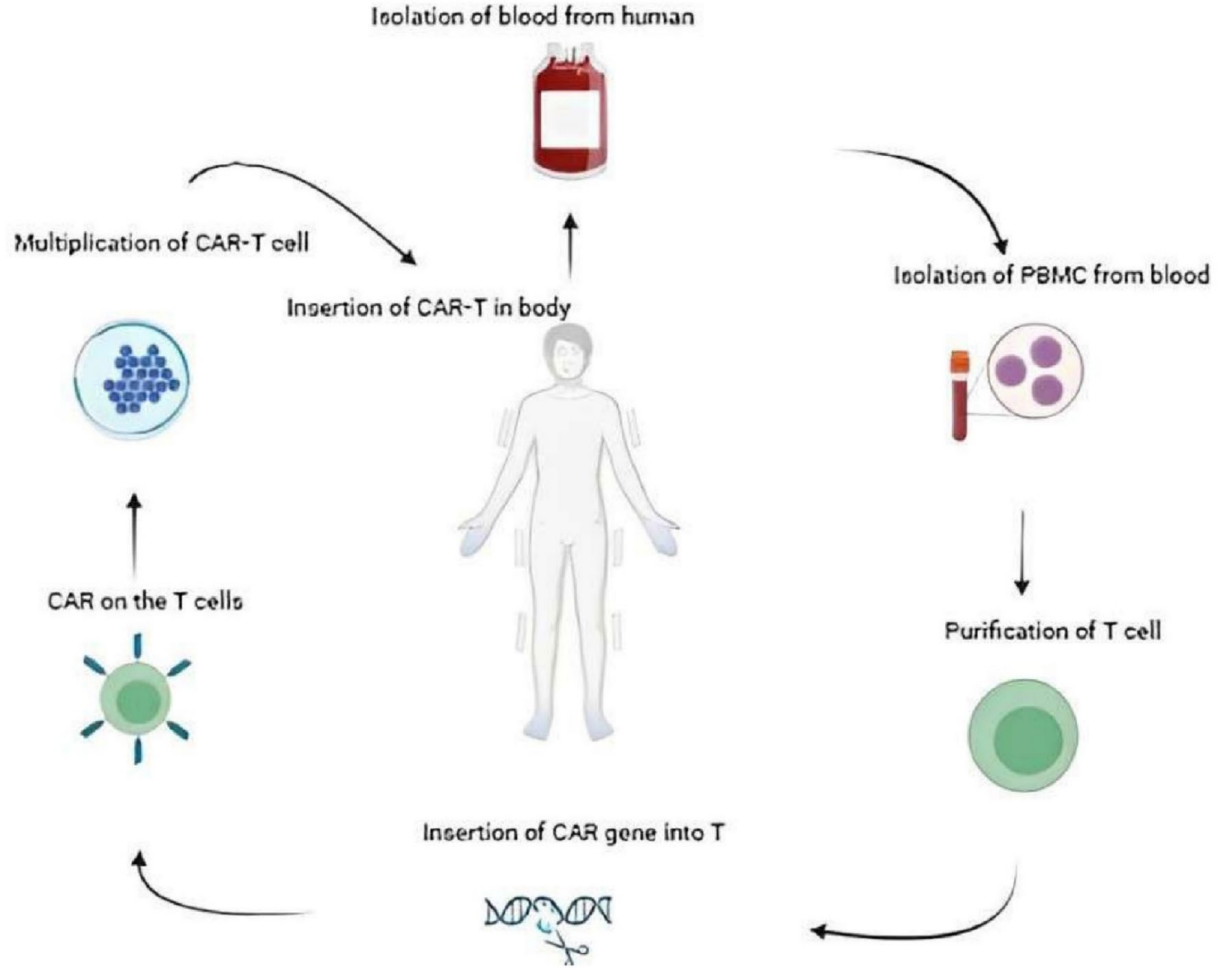
**“Ex-vivo generation of CAR-T cells, a traditional approach.”** For an autologous method of forming CAR-T cells, blood is drawn from humans, PBMCs are extracted from it, T cells are separated and purified. After inserting CAR gene in T cells, they start expressing that CAR on their surface. This colony of CAR T cells then multiplies in a culture environment until cells are inserted back into the donor

Traditionally, CAR T cells were manufactured by isolating T cells from the blood of a donor. A vector carrying the CAR sequence, in the form of scFv or mRNA, was introduced into the T cells. This sequence was translated in the T-cell nucleus and expressed on the cell surface. These CAR T cells were then multiplied ex vivo. Once enough CAR T cells were obtained, they were reintroduced into the patient's body (Fig. 2) [35].

**Fig. 3:**
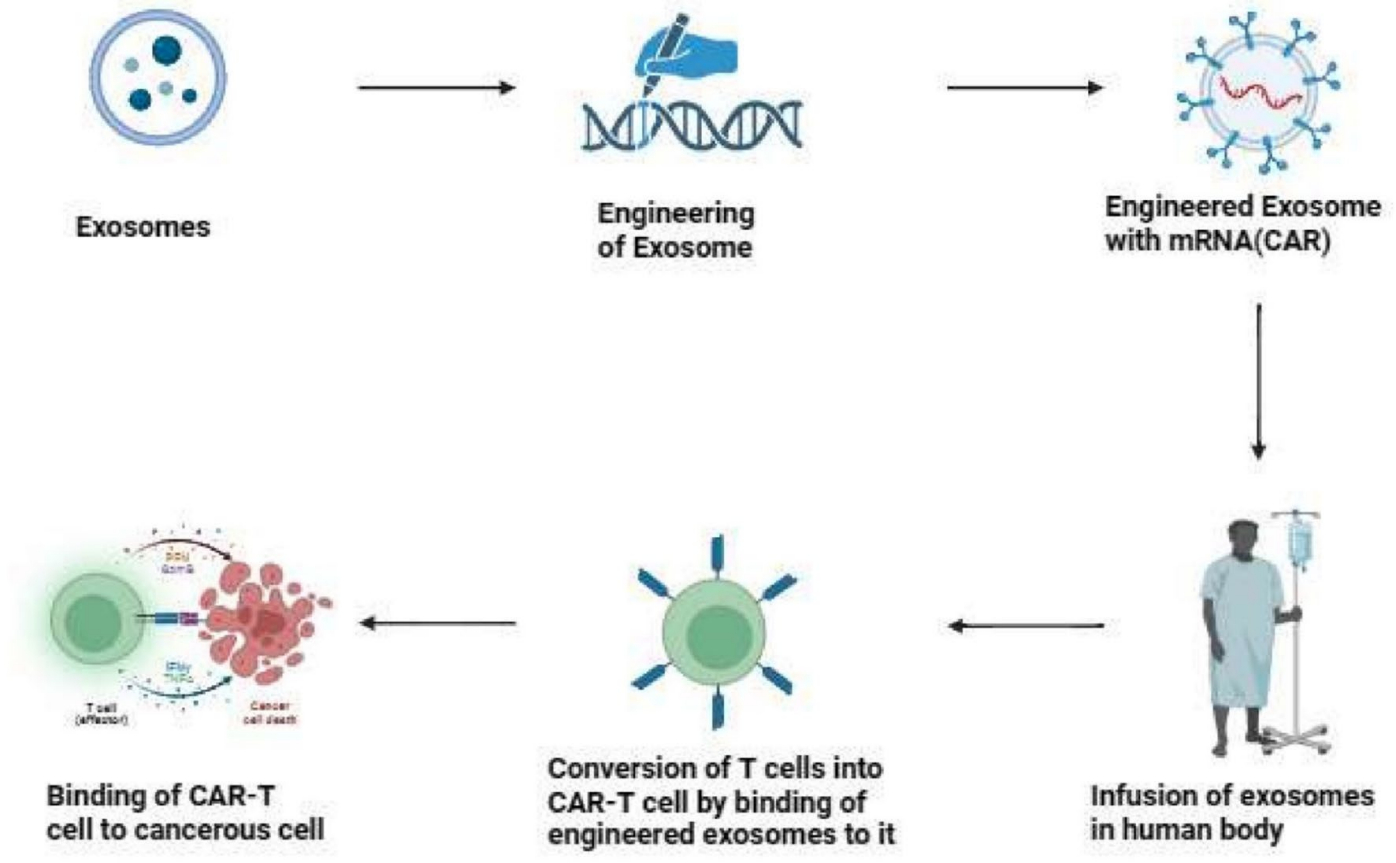
**“In-vivo generation of CAR-T cells, a gentle and modern approach.”** After engineered exosomes containing CAR labeled mRNA is infused in the human body, exosomes bind and enter the T cells, because of introduction of CAR labeled mRNA in the T cells, they became CAR T cells which then perform their function by binding and attacking cancerous cells

In recent advancements, the researchers are specifically targeting T cells by their receptors. They deliver the mRNA carrying the CAR sequence directly into these cells. This strategy achieved great success in CAR-T cell therapy. Various cargos, such as specially designed nanoparticles and engineered exosomes, are used to deliver the CAR gene directly to T cells (Fig. 3) [36]. These cargos have peptides, antibodies, or proteins on their surface that target the CD3/28 receptors on T cells to activate them. Once inside the cell, mRNA translocates into the nucleus expressing the CAR on the surface of the targeted T cells [37].

## Limitations of CAR T-cell immunotherapy

**Fig. 4:**
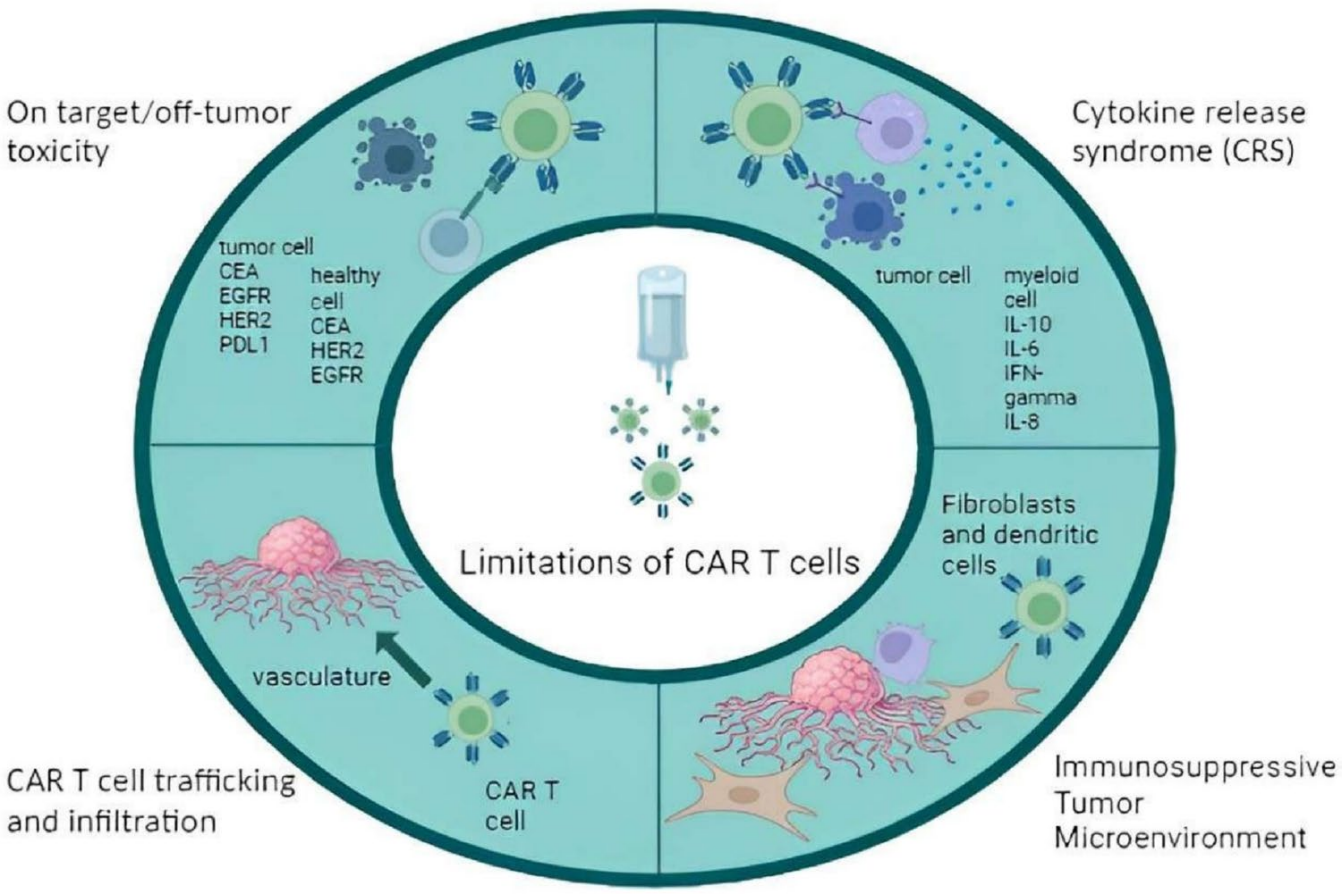
**“Limitations of CAR-T cells.”** There are limiting factors included in the failure of CAR T cells to perform their optimal functions. **a** On-target, off-tumor, toxicity (CAR T cells target the specific antigen, but it is also expressed on healthy cells). **b** CRS [a systemic inflammatory response). **c** CAR-T cells are unable to infiltrate the tumor vasculature, and **d** immune suppressive tumor microenvironment

KYMRIAH™ for acute lymphoblastic leukemia (ALL) and diffused B-cell lymphoma (DLBCL) as well as YESCARTA™ are CAR-T cell therapies which are clinically approved. Despite FDA approval, these CAR-T products can cause complications, which are generally manageable with appropriate therapeutic combinations and palliative care [38]. Beyond structural compromises in CAR-T cells products, significant challenges in the form of immunotherapy include tumor microenvironment barriers and immunosuppressive factors [39] (Fig. 4).

### On-target off-tumor toxicity

On-target off-tumor toxicity is a significant limitation of CAR T-cell therapy. It occurs when normal tissues expressing the targeted antigen are attacked by CAR T cells [40]. In a clinical trial for metastatic (RCC), carbonic anhydrase IX (CAIX) CAR-T cells caused hepatic toxicity by infiltrating CAIX-expressing bile duct epithelium, leading to the trial's termination. As a result, the trial was aborted [41].

### CAR T cells-related cytotoxicities

Tumor type, target antigen, and CAR design all influence the development and severity of CAR T cells-related toxicities. CAR T-cell-related toxicities include cytokine release syndrome (CRS), macrophage activation syndrome-like activation (MAS-L), and immune effector cell-associated neurotoxicity syndrome (ICANS) [42]. CRS, the most common toxicity, involves the release of interleukins (IL-2, IL-6, IL-8, and IL-10) and cytokines such as interferon gamma (IFN-γ), leading to systemic inflammatory response, vascular leakage, and multiorgan failure [33, 34]. ICANS may result from inflammation-induced disruption of the blood–brain barrier, causing encephalopathy, motor dysfunction, and potentially cerebral edema and even coma [43]. Hemophagocytic lymphocytosis (HLH) is a relatively rare condition. This condition is distinguished by fever, coagulopathy, hyper-ferritinema, spleen enlargement, hypertriglyceridemia, and cytopenia, the reason possibly being improper immune activation due to CAR T-cell immunotherapy [43].

### CAR T-cell trafficking & tumor infiltration

Peter pe et al. investigated that why CAR T cells show limited ability in treating solid cancers such as carcinomas (CA) and sarcomas (SARC) in comparison with liquid/hematological malignancies such as acute lymphoblastic leukemia (ALL) and chronic lymphocytic leukemia (CLL); he concluded that: physical barriers such as stroma of a solid tumor limit the capability of CAR T cells to traffic and infiltrate them. Local administration of CAR T cells to the site of tumor is a proposed solution compared to the traditional systemic delivery [44].

### Immune suppressive tumor microenvironment

Immunosuppressive cells including myeloid-derived suppressor cells (MDSCs), regulatory T cells (T-regs), and tumor-associated macrophages (TAMs) which infiltrate the cells inflicted with cancer contribute to immunosuppressive tumor microenvironment (TME) [45]. These tumors associated cells lead to the release of tumor-supporting chemokines and cytokines, an important factor causing the decline of natural antitumor immunity. CAR T cells as a result face poor T-cell expansion, CAR-T cell exhaustion, and a short persistence period [45]. There is a lot of evidence in support of IL cladded CAR T cells which express pro-inflammatory cytokines such as IL-12 and IL-15. As a result, immunosuppressive cytokine signaling (e.g., IL-4) is redirected toward pro-inflammatory signaling pathways [46] [47].

### Limitations of other CAR cells

Due to the limitations of CAR-T cells, CAR-NK (natural killer) and CAR-M (macrophage) cells have been developed and are showing promising potential for immunotherapy. CAR-NK cells are known for their cytotoxicity and have a rapid killing ability. There is an ongoing effort to design CAR-M cells due to their phagocytic properties. There is ongoing research to design effective and efficient CAR-M, leveraging their phagocytic properties. Some studies suggest that CAR-M cells maybe more effective at infiltrating the tumor microenvironment (TME) than CAR-T cells [48, 49].

However, CAR-NK and CAR-M also face limitations: CAR-NK cells are limited expansion ex vivo [50], and CAR-M cells do not proliferate either in vivo or ex vivo [51]. Consequently, this review focuses on strategies to enhance CAR-T cell immune therapy.

## Impact of tumor microenvironment on CAR-T cell therapy

Understanding the dynamics of tumor microenvironment (TME) is crucial for effective tumor management. CAR-T cell therapy has shown an early success in treating hematological malignancies such as leukemias and lymphomas. Reasons for this success include homogenous tumor microenvironment and easily accessible sites through hematogenous spread [52]. However, despite therapeutic promise, toxic complications including graft-versus-host disease and mortality are still major concerns [53].

For solid tumors, the TME is a complicated setting comprising cellular and acellular components [54]. The solid TME is both heterogeneous and complex. As these tumors grow, they transform into structures filled with connective tissue, vasculature, and extracellular matrix. Mechanical factors such as stiffness, solid stress, interstitial fluid pressure, and micro-architecture affect solid tumor severity and metastasis. The disrupted mechanical environment and biochemical profile of solid tumors thus hinders CAR-T cells from reaching the tumor sites effectively, leading to their therapeutic exhaustion and inefficiency [54–56] (Fig. 4).

## CAR-T cells in combination with immunomodulators

Both toxicity and therapeutic failure are counteracted by adopting combinatory therapies [57]. This review will focus on the role of eight immunomodulators to be used with CAR T-cell therapy for enhanced efficacy or to reduce potential toxicities. Research and investigation are rigorously being done to discover the immune modulatory effects of sulforaphane (a phytochemical) [58], as well as FDA-approved drugs such as sunitinib [58]*, *sorafenib [59]*, *Dasatinib [60], and metformin [61], in addition to their direct cancer curative effects. Furthermore, there is satisfactory evidence that interleukins such as IL-15 and IL-23 perform immune modulation and affect many other cancer-suppressing mechanisms [62, 63].

### Sulforaphane

Sulforaphane (SFN) is a phytochemical enriched in cruciferous vegetables such as broccoli and broccoli sprouts. It is chemically an isothiocyanate. SFN is a proven chemoprotective agent. SFN inhibits tumor development and cancer progression following multiple mechanisms [64]. Sulforaphane not only causes inhibition of tumor progression, but it is also said to be a modulator of immune cell physiology and differentiation potential [65].

**Fig. 5:**
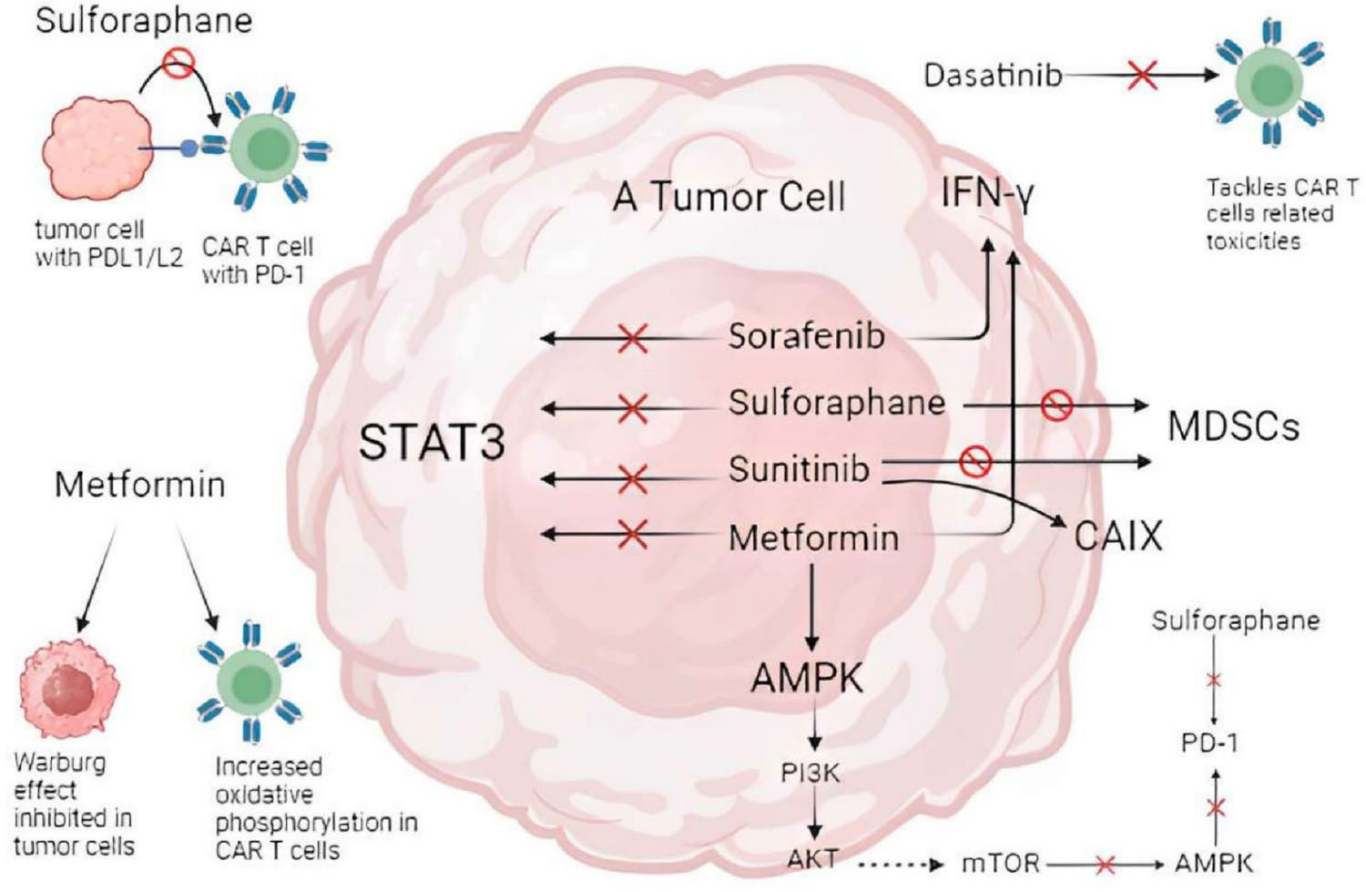
**“Pathways affected by drugs.”** Drugs acting as immunomodulators inhibit STAT3 signaling which engages in proliferation and metastasis. **a** Sulforaphane reduces the production of PD-L1 from cancer. **b** Dasatinib works to enhance CAR-T cell therapy, it reduces the chances of adverse toxic reactions. **c** Metformin inhibits the Warburg effect in tumor cells while increasing the rate of oxidative phosphorylation in CAR T cells

Shen et al. reported that SFN in the case of solid tumors inhibits PI3/AKT pathway followed by downregulation of PD-1 (programmed death receptor 1) in CAR T cells. It also causes PD-L1 (programmed death ligand 1) degradation in cancer cell via ubiquitination-mediated proteolysis pathway (Fig. 5). Research reported that control group SFN-treated CAR T-cell group produced higher levels of IFN-gamma, perforin, and granzyme B which result in increased cytotoxicity by CAR T cells. PI3K/AKT signaling pathway engages in differentiation and survival of T cells, so SFN manipulates PI3/AKT axis. SFN regulates PD-1 expression partially via PI3/AKT pathway. This predicted that SFN holds the capacity to attenuate the production of PD-L1 produced by tumor cells in response to IFN-gamma exposure. Clinical trials were conducted, they evaluated the effectivity of this combinatory with CAR T cells, patients received SFN orally, PD-1 and IFN-gamma expression was measured in CD8 + CAR-T cells in the peripheral blood drawn at separate times.

The results conclude that in SFN-treated subjects, PD-1 levels were decreased; whereas, IFN-gamma and IL-2 expressions were increased [66] [67]. Outcome of the research investigating sulforaphane as a naturally occurring immunomodulator is that sulforaphane has more potent affects than PF-04691502 (a synthetically produced kinase inhibitor) in downregulating downstream cellular activity of PI3K/AKT including p-AKT, p-mTOR, and p-S6. This leads to enhanced functionality of Meso-CAR T cells after in vitro treatment of SFN.

### Sunitinib

Sunitinib, a small-molecule tyrosine kinase inhibitor, targets tumor angiogenesis and associated receptors, including vascular endothelial growth factor (VEGF) receptor. Approved by the FDA for RCC treatment in 2006, sunitinib has shown immunostimulatory effects [67]. Its synergy with CAR T cells arises from upregulated CAIX expression in renal tumor cells and reduced myeloid-derived suppressor cells (MDSCs) frequency in the tumor microenvironment. Sunitinib enhances CAIX expression under both hypoxic and normoxic conditions, aiding CAR T-cell proliferation [68, 69].

Sunitinib is an excellent counterpart for combinatory approach in CAR T cells therapy in vitro. In murine lung metastatic models of human renal cancer, sunitinib mechanistically increases the frequency of CAIX CAR T cells in the peripheral blood and tumor cells. The possible mechanism behind this is that sunitinib increases the expression of CAIX on the tumor cells, which mediates the proliferation of CAR T cells in murine models receiving combinatory therapy. Sunitinib also regulates myeloid-derived suppressor cells (MDSCs) by causing the inhibition of STAT3 signaling. Apoptosis of MDSCs in vivo results which causes a shift in the activation and recruitment of immune cells and overall remodeling of tumor microenvironment in the favor of the CAR T cells therapy.

Although promising in vitro and in vivo, further research is needed [69]. Success mainly lies in the fact that this combination targets tumor vasculature, which supports the CAR T cells in their accessibility to the tumor site [70].

### Sorafenib

Sorafenib, a multi-kinase inhibitor, targets VEGFR, platelet-derived growth factor receptor (PDGFR), receptor tyrosine kinase type III (KIT), FMS‐like tyrosine kinase 3 (FLT-3), and receptor tyrosine kinase (RET). It is the standard treatment for advanced hepatocellular carcinoma (HCC) and is FDA-approved as the first-line systemic therapy. Sorafenib has immune modulatory effects, activating AMP-activated protein kinase (AMPK), a tumor suppressor (Fig. 5) [71].

Sorafenib in combination with other immunotherapies including toll-like receptor 3 (TLR3) agonists, dendritic cell therapy, and promises great advances in CAR T-cell therapy [71]. Other than that, a combination of other tyrosine kinase inhibitors included, and some immune checkpoint inhibitors are also in their phase three clinical trials as nominees to treat advanced hepatocellular carcinoma along with CAR T-cell therapy as a combinatorial approach [72]. This sort of supplementary therapy has shown noticeable reduction in tumor size in the pre-clinical HCC models. Sorafenib enhances antitumor responses by enhancing the functionality of tumor-specific effector T cells, while reducing the frequency of immune suppressor cells including T regulatory cells (T-regs). Wu et al. defends in his study that sorafenib at sub-pharmacological 5-Mm dose showed excellent apoptosis-inducing effects on HCC cell line Hepa1-6chGPC3 in combination with mesothelin CAR-T (m-CAR T) cell treatment. The study by Wu et al. suggests that sorafenib promotes antitumor response by the following mechanisms: 1—macrophage modulation and 2—triggering of apoptosis. Macrophagic modulation was made possible by the upregulation of interleukin-12 (IL-12) in the macrophages by the treatment of sorafenib. This exhibited immune stimulatory function. Low doses of sorafenib also enhanced the release of IFN-γ by m-CAR T cells [73].

In short, sorafenib promotes apoptosis, proliferation, and angiogenesis of the tumor cells by inhibiting intracellular serine–threonine kinases and multiple cell surface tyrosine kinases in the downstream [73].

### Dasatinib

Dasatinib is a small-molecule inhibitor of tyrosine kinases, dasatinib is considered a drug of superior integrity because its pharmacokinetics are not affected by age, race, and renal insufficiency [74]. It approved by the FDA in 2006 for the treatment of chronic myeloid leukemia and Philadelphia-positive chronic lymphoblastic leukemia. It is one of a kind drug for which low doses produce better results in terms of efficiency and reduced chances of toxicity [75].

Dasatinib enhances CAR T-cell therapy by reversibly inactivating CAR T cells during adverse lethal toxic reactions [75] (Fig. 5). CAR T cells are the “living drugs” which can get out of control at times. They can persist in patients for several years and can be subjected to sequential expansion and re-expansion after every exposure to the antigen. Therefore, the chances of on-target off-tumor activation are numerous. In cases of unrequired activation, dasatinib can cause a halt in CAR T-cell activation without affecting viability so the therapy can be resumed later [76]. It effectively manages CRS (cytokine release syndrome) post-therapy. Dasatinib is also ranked higher in efficacy than dexamethasone, a steroidal drug. In the study of Weber et al. and Mastermann K et al., 100-nM dasatinib inhibited CAR T-cell function immediately, whereas dexamethasone caused delayed inhibition at doses as high as 100 μM [77]. After co-culturing CD19.28*ζ* or CD19.BB*ζ* CAR-T cells and dasatinib, escalated decrease in the CD69 and CD107a (activation and degranulation markers) was noticed [76]. Dasatinib causes a suppression ABL (a family of tyrosine kinases) and blocks CD3z and zeta chain-associated protein kinase 70 (ZAP70) which are directly involved in T-cell receptor (TCR) signaling. Post-toxicity, CAR T cells regain function after dasatinib removal and a latency period [78]. In murine CRS models, dasatinib reduced mortality by lowering the levels of IFN-*γ*, tumor necrosis factor (TNF-*α*), granulocyte macrophage colony-stimulating factor (Gm-CSF), and IL-2 [79].

Even after remarkable evidence, further pre-clinical and clinical investigation is necessary in support of this combinatorial treatment approach.

### Metformin

Metformin belongs to a class of biguanide drugs, primarily used for the treatment of diabetes mellitus type 2 [80]. As the years pass, evidence of metformin being an important anticancer and anti-aging drug is found. In terms of malignancies, it kills cancer and cancer-initiating stem cells via activating AMPK pathway, which has a downstream inhibitory effect on cellular proliferation kinases such as PI3/AKT/mTOR [81].

In this review, we are focusing on the action of metformin on immune cells and its use as a combinatory therapy along with CAR T-cell therapy. In the study of [82], Chao et al. metformin and CAR T cells containing hydrogels work synergistically to enhance the multiplication and functionality of CAR T cells and direct CAR T cells to distant tumor sites. In human HGC-27 gastric carcinoma and human pancreatic murine models, Met/CAR T cells administration into the site of tumor, post surgically, ensured tumor cell clearance. Metformin modulates the Warburg effect and oxidative phosphorylation, inhibiting the glucose utilization by cancer cells while enhancing CAR T-cell respiration and ATP production by utilizing AMPK [Fig. 5]. Hydrogel vehicles for co-administration allow slow and constant release, improving tumor infiltration [81].

Although this study is a success, contrary research explains that metformin may lead to reduced proliferation of CAR T cells via AMPK pathway. This immunomodulatory effect of metformin can be utilized during unrequired and unsettling persistence of CAR T cells which leads to uncontrolled immune activation, cytotoxicity, and potential life-threatening side effects [81].

### IL-23

**Fig. 6:**
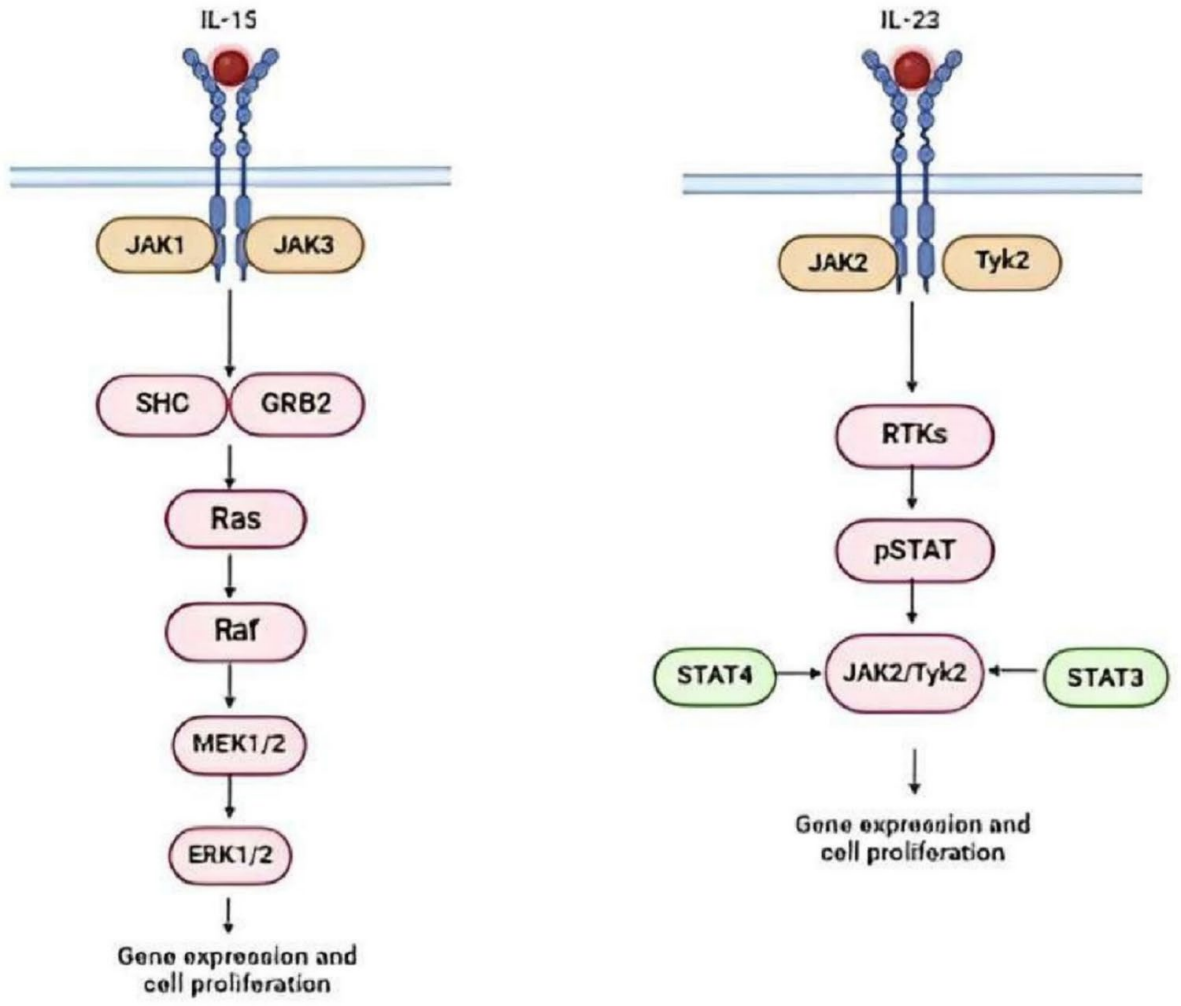
**“Pathways affected by Interleukins.” a** Interleukin-15 (IL-15) activates Janus kinases (JAK1 and JAK3). This leads to the downstream activation of SHC and GRB2 which activate Ras-Raf-MEK-ERK cascade. As a result, ERK1/2 translocate to the nucleus and regulate gene expression of various cellular processes, including cell proliferation. **b** Interleukin-23 (IL-23) activates JAK2 and Tyk2. This results in the recruitment of STAT proteins that regulate gene expression and immune responses such as T-cell proliferation and cytokine production

Interleukin-23 (IL-23) is a pro-inflammatory cytokine that combats cancer after binding to its specific receptor and activating the JAK/STAT pathway [83]. It also causes expansion of Th17 cells which are renowned for their antitumor activities [84]. Several types of activated immune cells such as CAR-T cells, dendritic cells, and macrophages are involved in the production of IL-23 [85] (Fig. 6).

Combining CAR T cells with IL-23 can improve immune cell infiltration at tumor sites and reduce suppressive environments [86]. They also play a significant role in eliminating cancerous cells from the body [87]. Ma et al. determined the effect of IL-23 in enhancing the function of IL-23 by constructing the plasmid for CAR-T cells with the P40-Td antigen activates the proliferation of IL-23 [88]. These CAR-T cells show the highest antitumor efficacy against solid tumors following CAR-T cell therapy in vivo and in vitro. STAT3-associated pathways are also activated by IL-23 [89].

### IL-15

Interleukin-15 (IL-15) is an inflammatory cytokine that addresses CAR-T cell therapy failures in solid tumors by enhancing T-cell proliferation through JAK/STAT3, PI3K, and MAPK pathways [90] [91] (Fig. 6).

In some cases, CAR-T cell therapy against solid tumors lacks persistence and shows exhaustion [92]. CAR T-cell therapy often lacks persistence and shows exhaustion, particularly against HCC due to insufficient antigen expression on cancer cells [93]. The expression of glypican-3 (GPC-3) on HCC cells provides a way to treat it by specifically targeting these receptors [94]. To treat patients with HCC, CAR-T cell therapy is under clinical trials that target GPC-3 [95]. GPC-3 is the oncofetal protein that is present during the development of the fetus, but later in adulthood, it is expressed as the oncogenic protein to develop the cancer in the adult human by activating the different pathways such as Wnt leads to the HCC. GPC-3 is also used as a diagnostic and prognostic biomarker for HCC [96].

To address this issue, Makkouk et al. designed CAR T cells with IL-15 induction sequences to enhance their efficacy against HCC. These modified CAR T cells showed superior immune responses compared to those lacking IL-15 [97].

## Conclusion

There is no doubt that CAR T-cell therapy is one of the promising immune therapies, which has shown notable results not only in terms of pre-clinical and clinical trials. It is also a certified treatment for hematological malignancies. For CAR T-cell therapy to produce significant clinical outcomes, many hurdles need to be crossed. Our review’s main aim was to evaluate the immunomodulators which, when given in a combination with CAR T cells, can lead to enhanced function and optimal persistence without making them overactive [98]. As the trend of immune therapies for cancer eradication is increasing, there is ever more need for the use of potential anticancer drugs and interleukins, which can modulate the immunotherapy for the best retrievable results. These drugs and interleukins enhance the efficacy of CAR T-cell therapy in comparison with the monotherapy approach. This is achieved via targeting pathways in a synergistic, antagonistic, or additive manner. One amazing result of using an integrative medicinal approach is that it reduces the chances of body getting resistant to the drugs or immune therapy if used alone [71]. Some aspiring effects which seem achievable by using immune modulatory agents are, aiding in reduction of tumor growth, removal of cancer stem cell populations, and induction of apoptosis in tumor cells [57].

Evidence supports that all the drugs and interleukins selected to be presented in this review showed enormous potential to be used in combinations [15, 67, 99]. Scientists are further evaluating the role of different cytokines and chemokines in cancer to introduce new treatment strategies [100]. Genes that regulate the expression of immunomodulators are transcribed by modified CAR-T cells, carrying specific sequences to enhance the immune response against cancerous cell [101]. It was only recently proposed that CAR T-cell trafficking to the site of tumor can be significantly improved by engineering CAR T cells that express chemokine receptors which counteract tumor producing chemokines.

In this review, several studies are summarized which aimed at regulating CAR T-cell function, differentiation, and persistence. They provide a guiding light to deal with recurrence, toxic reaction, and resistance related problems seen in cancer patients after CAR T-cell therapy. The provided combinatory approaches using drugs and interleukins (ILs) will help formulate ways to expand the horizons of CAR T-cell therapy, beyond hematological malignancies, pre-clinical models, and clinical trials to treat solid tumors.

## Abbreviations

CAIX: Carbonic anhydrase IX
CAR: Chimeric antigen receptor
CD3*ζ*: Cluster of differentiation 3 zeta
CoS: Costimulatory domains
CRS: Cytokine release syndrome
FcR*γ*: Fragment crystallizable receptor gamma
GMCSF: Granulocyte monocyte colony-stimulating factor
GPC-3: Glypican-3
HGC: Human gastric carcinoma
ICANS: Immune effector cell-associated neurotoxicity syndrome
IFN: Interferon
IL: Interleukin
ITAMs: Immune receptor tyrosine-based activation motifs
MDSCs: Myeloid-derived suppressor cells
mTOR: Mechanistic target of rapamycin
nM: Nanomolar
PBMCs: Peripheral blood mononuclear cells
PDGFR: Platelet-derived growth factor receptor
PD-L1: Programmed death ligand 1
PI3K/AKT: Phosphoinositide 3-kinase/protein kinase B
RCC: Renal carcinoma
SFN: Sulforaphane
TAA: Tumor-associated antigen
TCR: T-cell receptor
TME: Tumor microenvironment
TRUCKs: T cells redirected for universal cytokine-mediated killing
T-regs: T regulatory cells
VH: Variable heavy chain
VL: Variable light chain
ZAP70: Zeta chain-associated protein kinase
